# Clinical and epidemiological characterization of severe *Plasmodium vivax* malaria in Gujarat, India

**DOI:** 10.1080/21505594.2020.1773107

**Published:** 2020-06-03

**Authors:** Anupkumar R. Anvikar, Anna Maria van Eijk, Asha Shah, Kamlesh J. Upadhyay, Steven A. Sullivan, Ankita J. Patel, Jaykumar M. Joshi, Suchi Tyagi, Ranvir Singh, Jane M. Carlton, Himanshu Gupta, Samuel C. Wassmer

**Affiliations:** a Indian Council of Medical Research (ICMR), National Institute of Malaria Research, New Delhi, India; b Indian Council of Medical Research (ICMR), National Institute of Malaria Research Field Unit, Civil Hospital, Nadiad, India; c Center for Genomics and Systems Biology, Department of Biology, New York University, New York, NY, USA; d Byramjee Jeejeebhoy Medical College (BJMC), Civil Hospital, Ahmedabad, India; e Department of Infection Biology, Faculty of Infectious and Tropical Diseases, London School of Hygiene and Tropical Medicine, London, UK

**Keywords:** Severe malaria, *Plasmodium vivax*, Gujarat, India, malaria elimination

## Abstract

The mounting evidence supporting the capacity of *Plasmodium vivax* to cause severe disease has prompted the need for a better characterization of the resulting clinical complications. India is making progress with reducing malaria, but epidemics of severe vivax malaria in Gujarat, one of the main contributors to the vivax malaria burden in the country, have been reported recently and may be the result of a decrease in transmission and immune development. Over a period of one year, we enrolled severe malaria patients admitted at the Civil Hospital in Ahmedabad, the largest city in Gujarat, to investigate the morbidity of severe vivax malaria compared to severe falciparum malaria. Patients were submitted to standard thorough clinical and laboratory investigations and only PCR-confirmed infections were selected for the present study. Severevivax malaria (30 patients) was more frequent than severe falciparum malaria (8 patients) in our setting, and it predominantly affected adults (median age 32 years, interquartile range 22.5 years). This suggests a potential age shift in anti-malarial immunity, likely to result from the recent decrease in transmission across India. The clinical presentation of severe vivax patients was in line with previous reports, with jaundice as the most common complication. Our findings further support the need for epidemiological studies combining clinical characterization of severe vivax malaria and serological evaluation of exposure markers to monitor the impact of elimination programmes.

## Introduction

*Plasmodium vivax*(*Pv*) is the most geographically prevalent human malaria parasite, with 2.5 billion individuals at risk of infection worldwide [1]. Historically, *Pv* infection has been perceived as relatively benign, despite being debilitating and often life-threatening. As a result, clinical malaria research has mainly focused on *Plasmodium falciparum*(*Pf*) morbidity and mortality to date [2,3]. However, *Pv*has now become the predominant cause of malaria outside sub-Saharan Africa and is considered a key obstacle to malaria elimination [4]. This is particularly relevant in India, which remains the largest contributor to *Pv* burden globally [5,6], and where there has been an increase in reports supporting the capacity of *Pv* to induce severe and potentially fatal malaria in recent years [7–11]. This may be attributed to a combination of several factors, including historical underreporting, improved diagnostic granularity, and the availability of molecular tools to accurately differentiate parasite species and potential co-infections, or drug resistance emergence [10].

Gujarat is one of the major states contributing to the *Pv* burden in India, with a *Pv*/*Pf* ratio around 80:20 [12]. Remarkably, about one in five malaria-associated deaths reported in that state has been attributed to *Pv* [12,13]. This is in line with the mounting evidence for the ability of *Pv*to induce a wide spectrum of severe malaria, with a risk of death similar to that observed for *Pf* infection [3,7,8,10,11]. *Pv* invades primarily reticulocytes and uses the Duffy antigens expressed on the red blood cells as a receptor [3,14,15]. It is often found circulating at low parasite densities in the peripheral blood and has the ability to produce hypnozoites, or dormant liver stages, which lead to recurrent infections [3]. Several reports have shown the presence of *Pv* stages in the spleen, lungs, and bone marrow [16–20], suggesting that contrary to another common belief, *Pv*-infected reticulocytes may sequester and therefore play a role in the development of severe *Pv*malaria.

Endemic areas differ widely in terms of transmission, population immunity, and resulting severe disease frequency, prompting the need for a more comprehensive characterization of the spectrum of clinical complications in India. We conducted a prospective study to follow severe malaria patients with PCR-confirmed diagnosis admitted to the Ahmedabad Civil hospital in Gujarat, India, over a one-year period (December 2016 – November 2017), with the aim to meticulously differentiate and compare clinical and laboratory characteristicsbetweensevere *Pv*and*Pf*malaria infections.

## Materials and methods

### Study site

The study was set up in the state of Gujarat, which has the third highest *Pv* malaria burden in the country, accounting for 9% of the national total [12]. Patients were enrolled at the Civil Hospital in Ahmedabad, the largest city in Gujarat with an extended population of 6.3 million. Affiliated with the ByramjeeJeejeebhoy Medical College, the hospital is the largest in Gujarat, with 4,800 inpatient beds, and over 100,000 admissions per year. It is the main referral hospital for the adjoining districts of Mehsana, Gandhinagar, Kheda, Anand, Botad, Bhavnagar, and Surendranagar, and has facilities for dialysis and ventilation. *Anopheles culicifacies, An. stephensi,* and *An. fluviatilis* are the major malaria vectors in the region, with transmission closely following the monsoon, from mid-June to mid-October [12].

### Study design and patient selection

This prospective, hospital-based study focused on patients admitted with severe*Pf, Pv*or *Pf/Pv* co-infections, and was conducted between December 2016 and November 2017. Microscopy-diagnosed *Pf* and *Pv* infections with or without WHO-defined severe malaria symptoms [21,22] (Table 1), as well as one case of suspected malaria but with negative microscopy results, were enrolled after informed consent forms were obtained from patients or their legal representatives. 200μlof packed red blood cells was collected and used for PCR confirmation of *Plasmodium* species (*Pf, Pv, P. malariae, P. ovale)*, and only PCR-confirmed cases were selected for the subsequent analyses.

**Table 1.** Criteria used for the definition of severe malaria.

Clinical features	Description
Impaired consciousness	Including unarousable coma Assessment by Glasgow scale (10 or less) or Blantyre scale (3 or less) Normal cerebrospinal fluid
Prostration	Generalized weakness so that the patient is unable to sit, stand, or walk without assistance
Multiple convulsions	Jerky limb movements and staring eyes; more than two episodes within 24 hours
Deep breathing and respiratory distress	Acidotic breathing, arterial pH <7.35
Acute pulmonary oedema and acute respiratory distress syndrome	Tachypnea, dyspnea, and bilateral basal rales
Circulatory collapse or shock	Systolic blood pressure <80 mm Hg in adults, and <50 mm Hg in children
Acute kidney injury	Urine output <400 ml/24 hours in adults and <0.5 ml/kg in children
Clinical jaundice plus evidence of other vital organ dysfunction	Serum bilirubin > 3 mg/dl
Abnormal bleeding	Spontaneous bleeding at gums, nose, venipuncture sites, gastrointestinal tract, blood tests suggestive of disseminated intravascular coagulation
**Laboratory**	
Hypoglycemia	<2.2 mmol/l or < 40 mg/dl. Anxiety, sweating, palpitation, dilatation of pupils, breathlessness, convulsions, alteration of consciousness
Metabolic acidosis	Plasma bicarbonate < 15 mmol/l
Severe normocytic anemia	Hemoglobin,5 g/dl or packed cell volume < 15% in children, hemoglobin <7 g/dl or packed cell volume <20% in adults
Hemoglobinuria	Urine is positive for hemoglobin
Hyperlactatemia*	Lactate > 5 mmol/l
Renal impairment	Blood urea > 20 mM
Pulmonary oedema	Radiological: bilateral infiltration in the lungs on chest film
Hyperparasitemia	Parasite density > 100,000/µl (~ 2.5% parasitemia), appearance of peripheral schizontemia

*Not measured in our cohort.

### Clinical assessment and treatment

The clinical team performed a physical examination at the time of admission, and patients with suspected malaria infection were tested for *Pf* and *Pv* by microscopy and subsequently by PCR. Blood count, biochemistry analyses, chest X-rays, blood culture, and serology tests were also performed if deemed necessary by the physician in charge. *Pv*- or *Pf*-positive patients were then approached for enrolment and an additional 5 ml of blood was collected from enrolled individuals to evaluate potential co-infections. Clinical data were compiled in a case report form, which also included a history of previous malaria infections and the use of antimalarials, as well as pregnancy status for women of reproductive age. Treatments for *Pf*, Pv [23] and other co-infections (such as typhoid, dengue, chikungunya, tuberculosis, and viral hepatitis infections) were provided according to national guidelines of India. Clinical assessments and routine microscopy were performed on a daily basis until the patient recovered/died. Additional and follow-up laboratory tests were performed at the physician’s discretion, including serology tests and blood culture.

### Malaria diagnosis

Thick and thin microscopy blood smears were air-dried, stained with Giemsa, and examined using a light microscope. Blood samples were subjected to DNA extraction using the Qiagen® QIAamp DNA Midi Kits followed by PCR amplification to assess the presence of *Plasmodium* species based on the protocol described elsewhere [24–26]. Briefly, 25 μl of PCR reactions were prepared including 5 μl of template DNA, primers, and 1x master mix (GoTaq Green, Promega), reaction volume was raised by PCR-grade water. A BioRad T100 thermal cycler was used for amplification, and PCR products were run on 1.5% agarose gels (Agarose LE, Promega) in 1× TBE buffer (Thermo Scientific) to determine the presence of the amplified DNA, and PCR products were visualized using a UV trans-illuminator (BioRad Gel Doc EZ System). Different *Plasmodium* species were identified based on the amplified product sizes, as previously described [24–26].

### Ethical statement

Institutional Review Board approval was obtained from New York University and Institutional Ethical Clearance from NIMR. Informed consent was obtained from all subjects or, if subjects were under 18, from a parent and/or legal guardian. All laboratory tests were performed in accordance with the relevant guidelines and regulations.

### Dataset generation and statistical analysis

All case record forms (CRFs) were anonymized, digitized, and stored using the REDCap (Research Electronic Data Capture) system, which allowed subsequent extraction of datasets [27]. Chi-squared and Mann–Whitney U tests were used to compare categorical and continuous variables, respectively. Odds ratio (OR), 95% confidence interval (95% CI), and p-value were calculated using an online version of MedCalc software (https://www.medcalc.org/calc/odds_ratio.php). A two-sided p < 0.05 was considered statistically significant. All statistical analyses were performed using GraphPad Prism 8.0 (GraphPad Software).

### Data deposition

The study data are available for download through the Clinical Epidemiology Database (ClinEpiDB) [28] at https://clinepidb.org.

## Results

### Patients

**Figure 1.**
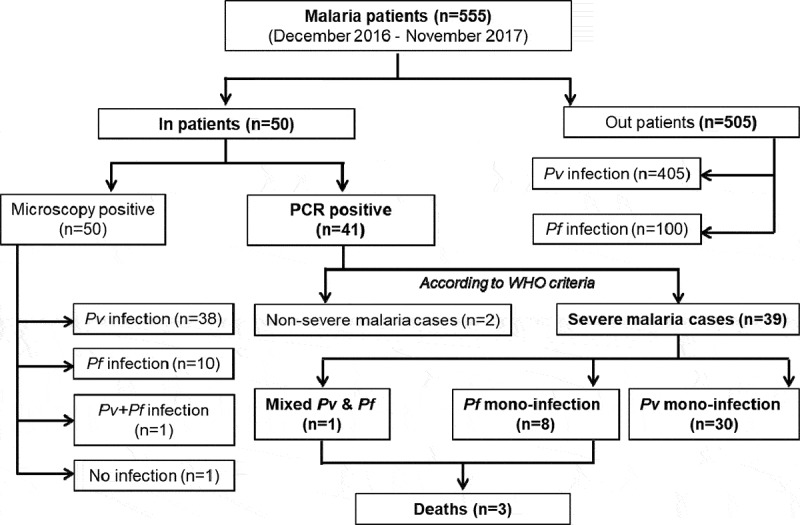
Flowchart of the study: of the initial 50 patients enrolled in the study, 9 were negative using PCR. Two did not match the WHO criteria for severe malaria, and of the 39 severe malaria cases, 30 were infected with *P. vivax* (*Pv*), 8 with *P. falciparum* (*Pf)*, and one with both parasite species.

Forty-one out of 50 enrolled patients were *Plasmodium* positive and diagnosed using PCR amplifications either for *Pf, Pv,* or *Pf*/*Pv* co-infection. No study participants were found positives for *P. malariae* or *P. ovale* infections. Thirty-nine patients had WHO-defined severe malaria [22]and two patients had non-severe malaria (Figure 1). Four patients were 15 years of age or below.Thirty patients had severe *Pv* infection, with a median age of 33.5 years (± 23.5), and a male to female ratio of 16:14 (Table 2). Eight patients had severe*Pf* infection, with a median age of 28.0 years (± 26.0), and a male to female ratio of 5:3. Only one patient had severe *Pf*/*Pv* co-infection (Figure 1). The odds of being admitted to the hospital with a diagnosis of *Pv* relative to *Pf* were OR = 0.94; 95%CI (0.45–1.95); p = 0.864. *Pv* patients had significantly higher sexual and asexual parasitemia compared to *Pf*, and *Pf* patients had wider variations in white blood cell counts (Suppl. Figure 1). Thrombocytopenia, a common complication of *Pf* and *Pv* malaria [29], was observed in both groups (Suppl. Figure 1); 84.6% and 41% of all severe malaria patients used public tanks or pumps as the water source and were below the poverty line, respectively; 43.6% and 25.6% of them lived in the thatch houses and worked as daily labours for their survival, respectively. The majority of the study participants (78%) were from Ahmedabad city, 17.1% of the patients were from Vadodara, Radhanpur, Surendranagar, Gandhinagar, Kheda, and Mehsana districts, and two (4.9%) patients were from Rajasthan. There was no significant difference between *Pv* and *Pf* infection when these demographic, socioeconomic, and epidemiological characteristics were compared. Ten severe malaria patients required blood transfusion, 7 out of 10 were infected with *Pv* parasites. Severe malaria *Pf* patients required assisted breathing (n = 3), dialysis (n = 1) and nasogastric tube (n = 2). The patient with both *Pf*/*Pv* co-infection presented complaints such as headache, chills, pains, vomiting, dizziness, elevated heart rate (96 beats/minutes) as well as increased bilirubin (24.5 mg/dL), urea (102 mg/dL), and creatinine (4.87 mg/dL) levels. Blood cultures were negative for all but one patient, who tested positive for *Bacillus subtilis* but was not categorized as having sepsis due to the individual’s clinical condition and immunocompetence. G6PD deficiency was only tested when deemed necessary by the physician, after asking the patient for any known blood disorders.6 *Pv* patients were tested for G6PD deficiency, and only one individual was positive.

**Table 2.** Demographic, socioeconomic, and epidemiological characteristics of severe malaria patients at the time of admission. Unless otherwise stated, values are expressed in the number of patients; percentages are in brackets. P-values were obtained by Chi-square or Mann–Whitney U tests; NA: not applicable; IQR: interquartile range; *Pv: P. vivax; Pf: P. falciparum.*

		*Pv* (n = 30)	*Pf* (n = 8)	p Value
Female		14 (46.7)	3 (37.5)	0.6431
Age, y, median±IQR		33.5 ± 23.5	28.0 ± 26.0	0.5319
Education status				
	Primary school	19 (63.3)	5 (62.5)	0.3412
	Secondary school	6 (20.0)	3 (37.5)	
	None	5 (16.7)	0	
Occupation				
	Daily labour	9 (30.0)	1 (12.5)	0.3004
	Salary service	7 (23.3)	4 (50.0)	
	Other	14 (46.7)	3 (37.5)	
Annual income				
	Above poverty line	20 (66.7)	3 (37.5)	0.1337
	Below poverty line	10 (33.3)	5 (62.5)	
House structure				
	Brick/concrete/other	16 (53.3)	5 (62.5)	0.6431
	Thatch	14 (46.7)	3 (37.5)	
Water source				
	Bore well	3 (10.0)	2 (25.0)	0.2648
	Public tank/pump/other	27 (90.0)	6 (75.0)	
Malaria history				
	Yes	5 (16.7)	1 (12.5)	0.774
	No	25 (83.3)	7 (87.5)	
Co-infections				
	Yes	6 (20.0)	0	0.1681
	No	24 (80.0)	8 (100.0)	
Pregnancy				
	Yes	2 (14.3)	0	0.4858
	No	12 (85.7)	3 (100.0)	
Weight, kg, median±IQR		60.0 ± 13.3	61.0 ± 17.0	0.9648
Height, cm, median±IQR		160.0 ± 11.8	164.0 ± 18.3	0.4641
Heart rate, beats/minutes, median±IQR		88.0 ± 15.0	84.0 ± 12.0	0.3601
Temperature °C, median±IQR		37.2 ± 0.4	37.2 ± 0.2	0.2798

### *Severe and non-severe* Plasmodium vivax *malaria*

Among 30 severe *Pv* cases, 16.7% (5/30) of the patients had history of fever or malaria in the previous 12 months and 20% (6/30) of them also had typhoid (n = 1), dengue (n = 1), chikungunya (n = 1), or tuberculosis (n = 1) co-infections; 23.3% (7/30) of patients came with fever (≥37.5°C). The median heart rate and body temperature were 88.0°C and 37.2°C, respectively. The demographic, socioeconomic, and epidemiological characteristics of these patients are presented in Table 2. Patients presented a variety of symptoms such as chills (100%), headache (96.7%), vomiting (76.7%), and abdominal pain (73.3%) (Table 3). Among the hematological parameters, platelet counts were recorded for only 80% (24/30) of patients, thrombocytopenia (<150,000 platelets/µL) was found in 58.3% of the patients (14/24), and 29.2% of the patients (7/24) presented severe thrombocytopenia (<50,000 platelets/µL). The hematological and biochemical parameters of these patients are presented in Table 4. Among severe *Pv* cases, prostration (n = 27) was the most prevalent of the severe malaria signs (Table 5), followed by multiple convulsions (n = 21), renal impairment (n = 12), and clinical jaundice (n = 10). Information on symptoms of the severity of the remaining patients is presented in Table 5. Two non-severe malaria patients had a better socioeconomic status, no history of fever or malaria in the previous 12 months, no co-infections, and better hematological and biochemical parameters. No *Pv* infected patients presented primaquine-associated hemolysis and related complications.

**Table 3.** Summary of clinical signs and symptoms of enrolled severe malaria patients. Values are expressed in the number of patients and percentages are in brackets. P-values were obtained by Chi-square test; NA: not applicable; *Pv: P. vivax; Pf: P. falciparum.*

	*Pv*	*Pf*	p Value
Headache	29 (96.7)	8 (100.0)	0.6007
Chills	30 (100.0)	8 (100.0)	NA
Aches or pains	16 (53.3)	3 (37.5)	0.4261
Fatigue	5 (16.7)	3 (37.5)	0.1991
Vomiting	23 (76.7)	7 (87.5)	0.5043
Lack of appetite	9 (30.0)	2 (25.0)	0.7817
Cough	9 (30.0)	4 (50.0)	0.2894
Dizziness	5 (16.7)	1 (12.5)	0.774
Sweating	1 (3.3)	2 (25.0)	0.0435
Abdominal pain	22 (73.3)	6 (75.0)	0.9242
Diarrhoea	3 (10.0)	1 (12.5)	0.8378
Lower back pain	4 (13.3)	1 (12.5)	0.9506
Eye pain	0 (0)	0 (0)	NA
Paleness	0 (0)	0 (0)	NA
Chest pain	2 (6.7)	0 (0)	0.4531
Rash	0 (0)	0 (0)	NA
Ear pain	0 (0)	0 (0)	NA
Splenomegaly	0 (0)	0 (0)	NA

**Table 4.** Hematological and biochemical laboratory findings of severe malaria patients. Values are expressed in median ± interquartile range; patient numbers are in brackets. P-values were obtained by Mann–Whitney U tests; NA: not applicable; *Pv: P. vivax; Pf: P. falciparum.*

	*Pv*	*Pf*	p Value
Hemoglobin levels (g/dL)	10.7 ± 3.8 (27)	8.1 ± 5.6 (8)	0.0568
Hematocrit (%)	33.9 ± 12.7 (27)	24.5 ± 19.5 (8)	0.0147
Platelet counts (x10^9^/L)	67.0 ± 72.8 (24)	42.0 ± 72.0 (7)	0.1492
Serum bilirubin (mg/dL)	2.7 ± 2.9 (22)	2.0 ± 1.7 (3)	0.3483
Serum creatinine (mg/dL)	0.8 ± 0.4 (22)	0.9 ± 9.4 (3)	0.1304
Blood urea (mg/dL)	25.2 ± 18.5 (22)	51.2 ± 87.9 (3)	0.107
ALT levels (U/L)	24.1 ± 64.6 (21)	72.0 ± 67.8 (3)	0.6196
Blood glucose (mg/dL)	127.0 ± 42.5 (5)	126.0 ± 74.8 (5)	0.9444
Sodium levels (mmol/L)	134.5 ± 10.7 (16)	130.3 ± 6.5 (2)	0.268

**Table 5.** Summary of symptoms of the severity of patients enrolled in the study according to WHO criteria of severe malaria. Values are expressed in the number of patients and percentages are in brackets. P-values were obtained by the chi-square test; NA: not applicable; *Pv: P. vivax; Pf: P. falciparum.*

Symptoms	*Pv*	*Pf*	p Value
Impaired consciousness	0 (0)	1 (12.5)	0.0497
Prostration	27 (90)	8 (100)	0.3514
Multiple convulsions	21 (70)	7 (87.5)	0.3179
Deep breath and respiratory distress	0 (0)	2 (25)	0.0049
Acute respiratory distress syndrome	0 (0)	0 (0)	NA
Shock	1 (3.3)	0 (0)	0.6007
Clinical jaundice	10 (33.3)	0 (0)	0.0571
Abnormal bleeding	1 (3.3)	0 (0)	0.6007
Hypoglycemia	0 (0)	0 (0)	NA
Metabolic acidosis	1 (3.3)	0 (0)	0.6007
Severe anemia	1 (3.3)	2 (25)	0.0435
Hemoglobinurea	1 (3.3)	0 (0)	0.6007
Hyperlactatemia	0 (0)	0 (0)	NA
Renal impairment	12 (40)	3 (37.5)	0.8977
Pulmonary edema	0 (0)	0 (0)	NA
Hyperparasitemia	0 (0)	0 (0)	NA

### *Severe* Plasmodium falciparum *malaria*

Out of 8 severe *Pf* cases, 12.5% (1/8) of the patients had a previous history of fever or malaria in the previous 12 months and no co-infections were found. Only one patient had a complaint of fever (≥37.5°C). The median heart rate and body temperature were 84.0°C and 37.2°C, respectively. The demographic, socioeconomic, and epidemiological characteristics of these patients are presented in Table 2. Patients complained of headache (100%), chills (100%), vomiting (87.5%), and abdominal pain (75%). Among the hematological parameters, platelet counts were recorded for only 87.5% (7/8) of patients, thrombocytopenia (<150,000 platelets/µL) was found in 42.9% of the patients (3/7), and 57.1% of the patients (4/7) presented severe thrombocytopenia (<50,000 platelets/µL). The hematological and biochemical parameters of these patients are presented in Table 4. Among severe *Pf*patients, prostration (n = 8) was the most prevalent of the severe malaria signs (Table 5), followed by multiple convulsions (n = 7) and renal impairment (n = 3). Two (25%) patients had severe anemia and 2 (25%) had deep breathing and respiratory distress. Details of severity symptoms for the remaining patients are presented in Table 5. Significant differences were observed for sweating (p = 0.043), hematocrit (p = 0.015), impaired consciousness (p = 0.049), deep breathing and respiratory distress (p < 0.01), and severe anemia (p = 0.043) when compared between severe *Pf*and *Pv*groups. However, clinically, these differences do not appear to be significant due to the small sample size. In addition, it is noteworthy that for the same reason, these differences are not significant once a correction for multiple testing is applied.

### Follow-up and severe malaria-associated deaths

All study participants (30 with severe *Pv*, 8 with severe *Pf*, 2 with non-severe *Pv*, and 1 with *Pf/Pv* co-infection) were admitted in hospital wards and were followed, treated, and evaluated until discharge, lasting for 3.39 days (± 2.13) in the hospital. No significant difference (p = 0.538) was observed for hospital admission duration between severe *Pf* (3.40 days ± 1.85) and *Pv* (3.86 days ± 3.14) patients; 78% (32/41) patients recovered, 14.6% (6/41) patients were discharged against the physician’s advice and 7.3% (3/41) patients died. Two of the fatal cases had *Pf* mono-infections and 1 *Pf*/*Pv* co-infection.

## Discussion

The benign nature of *Pv* infections has been widely challenged over the recent years, and studies using stringent diagnosis techniques demonstrated a similar risk of severe disease and death as with *Pf* [30].A comprehensive clinical characterization of acute *Pv* infection relying on molecular tools is pivotal to better understand the pathogenetic mechanisms leading to severe disease and inform not only clinical management but also new adjunct therapies. In endemic areas with low parasite densities and transmission where *Pv* is becoming the dominant species [31], the use of PCR-based assays is highly recommended as microscopy is not sensitive enough for routine malaria screening [9,11,32,33]. We therefore focused solely on PCR-positive patients in this study, as precise data on *Pv* burden is crucial to designing and implementing effective malaria control and elimination policies in India, where the Government has set an elimination target for 2030 [34].

We identified a higher frequency of severe *Pv* cases compared to *Pf*, which is consistent with the higher prevalence of the former species in the region [12]. The majority of study participants had a lower socioeconomic status and were from Ahmedabad; 29.2% of severe malaria patients presented severe thrombocytopenia. Prostration, multiple convulsions, renal impairment, clinical jaundice, severe anemia, and deep breathing and respiratory distress were prevalent among the symptoms of severity.

Jaundice was only observed in severe *Pv* cases, which aligns with data reported in previous studies, where it was the most common complication seen in severe vivax infections [9,35,36]. Hepatic injury causing jaundice and increased liver enzyme levels have also been associated with severe *Pf* infection [37–40]. However, elevated alanine aminotransferase (n = 6) and bilirubin (n = 10) levels in our cohort indicate that hepatic dysfunction was also present in study participants with severe *Pv* infection, suggesting that a combination of both hepatic dysfunction and hemolysis may be at play. One severe *Pv* patient had hemoglobinuria, but hypoglycemia was not detected in any of the enrolled patients. Although infrequent, hypoglycemia has been reported in severe *Pv* patients [9,41,42]. Severe *Pf* patients from our cohort presented two or more symptoms of severity on admission, while four or more were only seen in severe *Pv* patients. However, this did not affect the length of stay at the hospital. A high frequency of multiple convulsions was recorded in severe *Pv* cases. Because the occurrence of convulsions is not a WHO-defined criterion of severity for *Pv* infection, the history of epilepsy was not documented as part of our clinical protocol. A possible explanation is that these patients had a previous history of epilepsy, and convulsions were triggered by fever during *Pv* infection. Three patients enrolled in this study died. Two had *Pf* mono-infections and one *Pf/Pv* co-infection, indicating a higher fatality rate in*Pf*compared to *Pv*patients. This is in line with malaria-associated mortality data from the same hospital, which recorded a case fatality rate of 10.1% in *Pf*(43/425) compared to 5% in *Pv*(16/323) in 2011.

Severe*Pv* cases are treated with a 3-day course of chloroquine (25 mg/kg) followed by a 14-day course of primaquine (0.25 mg/kg). The use of primaquine prevents relapse by killing the dormant liver stages of *Pv*. However, the drug presents greater risks of hemolysis in G6PD-deficient patients [12,43,43]. G6PD deficiency is an X-linked inheritance, affecting 400 million people worldwide [43]. The overall magnitude of G6PD deficiency prevalence in India is considered to be high [44–46], with an estimated prevalence of G6PD deficiency ranging from 4.2% to 10.8% in Gujarat [12,47]. Following clinical assessments, six *Pv*patients enrolled in this study underwent G6PD deficiency testing, and only one individual was found positive. In accordance with the Indian national policy, all G6PD-negative,*Pv*-infected patients were treated with primaquine [23], and no side effects were observed in our cohort. While this may indicate that the use of primaquine in the local population could be safer than initially evaluated, systematic G6PD deficiency testing will be necessary to successfully implement radical cure regimens for *Pv* and support future malaria elimination efforts [48].

Socioeconomic factors have been shown to influence malaria case management, as they promote causal behaviors and choices [49,50]. In the present study, 41% (16/39) of participants were below the poverty line, 64.1% (25/39) only gained primary school education, 43.6% (17/39) were living in thatched houses, and 84.6% (33/39) of participants used public tanks/pumps as the water source. This indicates that the socioeconomic status of the majority of participants was low, which might have affected or delayed the decision on when and where to seek malaria treatment. Early and prompt treatment of severe malaria is critical in improving the prognosis, as any delay could lead to life-threatening outcomes. Previous studies have also associated lack of education, low income, and living in poorly constructed houses with increased risk of *Plasmodium* infection [51,52]. Therefore, efforts to control and eliminate malaria should also include investment in the socioeconomic development of malaria-endemic areas, which may have a substantial impact against malaria in the long term [51].

India has the world’s largest national malaria control program [34,53], which has led to a significant reduction in malaria transmission over the last decade [6]. In a study recently conducted by our group, almost all infections in both clinic and community surveys at another site in Gujarat were detectable by microscopy. Because the area has a history of low malaria prevalence, residents can be expected to have low immunity, which may explain why most infections will develop parasitemia not only detectable by microscopy but also will lead to clinical episodes [26]. The proportion of*Pv-*positive cases in this other site was 85.9% (61/71) and 83.3% (55/66) by microscopy and PCR, respectively [26], which aligns with 78% (32/41) of *Pv* cases reported in the present study. Collectively, these findings suggest that the recent decline in malaria transmission in India has not affected the overall ratio of *Pv: Pf* in Gujarat, which was reported to be 80:20 in 2014 [12]. However, the decrease of the development of naturally acquired immunity against the infection resulting from a reduction of transmission may lead to less total cases, but potentially an increase in severe cases [54]. Only one patient with severe *Pv* was under 15 years in our cohort, a proportion of 3.3% that is ten-fold lower than the 30% of the patients under 14 with severe *Pv* reported across India in 2016 [12]. Incidentally, the number of *Pv* cases in Gujarat has declined steadily since 2004, and the lack of exposure may explain this shift in the age of the patients with severe disease [12]. Our findings further support the need for epidemiological studies combining clinical characterization of severe *Pv* malaria and serological evaluation of exposure markers to monitor the impact of elimination programmes in the future.

In conclusion, severe malaria due to *Pv* was more frequent than severe *Pf* infection among patients admitted at Ahmedabad Civil Hospital, and it affected predominantly adults. Their clinical presentation was in line with previous reports, and the overall fatality rate due to severe malaria was lower than average (~7%). The present report contributes to our understanding of the disease spectrum of severe *Pv*malaria in India and is indicative of a high prevalence of severe *Pv* in Ahmedabad, which should be considered in local malaria elimination campaigns.

## Supplementary Material

Supplemental Material

## References

[1] Howes RE, Battle KE, Mendis KN, et al. Global epidemiology of Plasmodium vivax. Am J Trop Med Hyg. 2016;95:15–34. 10.4269/ajtmh.16-0141 PMC5198891 27402513

[2] Mackintosh CL, Beeson JG, Marsh K. Clinical features and pathogenesis of severe malaria. Trends Parasitol. 2004;20:597–603. 15522670 10.1016/j.pt.2004.09.006

[3] Mueller I, Galinski MR, Baird JK, et al. Key gaps in the knowledge of Plasmodium vivax, a neglected human malaria parasite. Lancet Infect Dis. 2009;9:555–566. 19695492 10.1016/S1473-3099(09)70177-X

[4] Carlton JM, Sina BJ, Adams JH. Why is Plasmodium vivax a neglected tropical disease? PLoS Negl Trop Dis. 2011;5:e1160. 21738804 10.1371/journal.pntd.0001160 PMC3125139

[5] Joshi H, Prajapati SK, Verma A, et al. Plasmodium vivax in India. Trends Parasitol. 2008;24:228–235. 18403267 10.1016/j.pt.2008.01.007

[6] WHO World Health Organization. World Malaria Report. 2018.

[7] Gupta H, Afsal MP, Shetty SM, et al. Plasmodium vivax infection causes acute respiratory distress syndrome: a case report. J Infect Dev Ctries. 2015;9:910–913. 26322886 10.3855/jidc.6813

[8] Gupta H, Dhunputh P, Bhatt AN, et al. Cerebral malaria in a man with Plasmodium vivax mono-infection: a case report. Trop Doct. 2016;46:241–245. 26748392 10.1177/0049475515624857

[9] Kochar DK, Das A, Kochar SK, et al. Severe Plasmodium vivax malaria: a report on serial cases from Bikaner in northwestern India. Am J Trop Med Hyg. 2009;80:194–198. 19190212

[10] Rahimi BA, Thakkinstian A, White NJ, et al. Severe vivax malaria: a systematic review and meta-analysis of clinical studies since 1900. Malar J. 2014;13:481. 25486908 10.1186/1475-2875-13-481 PMC4364574

[11] Siqueira AM, Lacerda MV, Magalhaes BM, et al. Characterization of Plasmodium vivax-associated admissions to reference hospitals in Brazil and India. BMC Med. 2015;13:57. 25889040 10.1186/s12916-015-0302-y PMC4404636

[12] Anvikar AR, Shah N, Dhariwal AC, et al. Epidemiology of Plasmodium vivax Malaria in India. Am J Trop Med Hyg. 2016;95:108–120. 27708188 10.4269/ajtmh.16-0163 PMC5201217

[13] Joshi U, Solanki A, Oza U, et al. Situation of P. vivax malaria in Ahmedabad city: A study in purview of national guidelines. Ann Trop Med Public Health. 2013;6:227–231.

[14] Mayor A, Alano P. Bone marrow reticulocytes: a Plasmodium vivax affair? Blood. 2015;125:1203–1205. 25700424 10.1182/blood-2014-12-614123

[15] Miller LH, Mason SJ, Clyde DF, et al. The resistance factor to Plasmodium vivax in blacks. The Duffy-blood-group genotype, FyFy. N Engl J Med. 1976;295:302–304. 778616 10.1056/NEJM197608052950602

[16] Anstey NM, Handojo T, Pain MC, et al. Lung injury in vivax malaria: pathophysiological evidence for pulmonary vascular sequestration and posttreatment alveolar-capillary inflammation. J Infect Dis. 2007;195:589–596. 17230420 10.1086/510756 PMC2532499

[17] Baro B, Deroost K, Raiol T, et al. Plasmodium vivax gametocytes in the bone marrow of an acute malaria patient and changes in the erythroid miRNA profile. PLoS Negl Trop Dis. 2017;11:e0005365. 28384192 10.1371/journal.pntd.0005365 PMC5383020

[18] Lacerda MV, Fragoso SC, Alecrim MG, et al. Postmortem characterization of patients with clinical diagnosis of Plasmodium vivax malaria: to what extent does this parasite kill? Clin Infect Dis. 2012;55:e67–74. 22772803 10.1093/cid/cis615

[19] Machado Siqueira A, Lopes Magalhaes BM, Cardoso Melo G, et al. Spleen rupture in a case of untreated Plasmodium vivax infection. PLoS Negl Trop Dis. 2012;6:e1934. 23272256 10.1371/journal.pntd.0001934 PMC3521714

[20] Obaldia N 3rd, Meibalan E, Sa JM, et al. Bone marrow is a major parasite reservoir in plasmodium vivax infection. mBio. 2018;9:e00625-18. 29739900 10.1128/mBio.00625-18 PMC5941073

[21] Organization WH. Guidelines for the treatment of malaria. 3rd ed. 2015.

[22] WHO. Severe malaria. Trop Med Int Health. 2014;19:7–131. 25214480 10.1111/tmi.12313_2

[23] Research NIoM. Guidelines for the diagnosis and treatment of malaria in India. 2011.

[24] Rubio JM, Benito A, Berzosa PJ, et al. Usefulness of seminested multiplex PCR in surveillance of imported malaria in Spain. J Clin Microbiol. 1999;37:3260–3264. 10488189 10.1128/jcm.37.10.3260-3264.1999 PMC85544

[25] Rubio JM, Benito A, Roche J, et al. Semi-nested, multiplex polymerase chain reaction for detection of human malaria parasites and evidence of Plasmodium vivax infection in Equatorial Guinea. Am J Trop Med Hyg. 1999;60:183–187. 10072133 10.4269/ajtmh.1999.60.183

[26] van Eijk AM, Sutton PL, Ramanathapuram L, et al. The burden of submicroscopic and asymptomatic malaria in India revealed from epidemiology studies at three varied transmission sites in India. Sci Rep. 2019;9:17095. 31745160 10.1038/s41598-019-53386-w PMC6863831

[27] Harris PA, Taylor R, Thielke R, et al. Research electronic data capture (REDCap)–a metadata-driven methodology and workflow process for providing translational research informatics support. J Biomed Inform. 2009;42:377–381. 18929686 10.1016/j.jbi.2008.08.010 PMC2700030

[28] Ruhamyankaka E, Brunk BP, Dorsey G, et al. ClinEpiDB: an open-access clinical epidemiology database resource encouraging online exploration of complex studies. Gates Open Res. 2019;3:1661. 32047873 10.12688/gatesopenres.13087.1 PMC6993508

[29] Lacerda MV, Mourao MP, Coelho HC, et al. Thrombocytopenia in malaria: who cares? Mem Inst Oswaldo Cruz. 2011;106(Suppl 1):52–63. 21881757 10.1590/s0074-02762011000900007

[30] Baird JK. Evidence and implications of mortality associated with acute Plasmodium vivax malaria. Clin Microbiol Rev. 2013;26:36–57. 23297258 10.1128/CMR.00074-12 PMC3553673

[31] Commons RJ, Simpson JA, Thriemer K, et al. Risk of Plasmodium vivax parasitaemia after Plasmodium falciparum infection: a systematic review and meta-analysis. Lancet Infect Dis. 2019;19:91–101. 30587297 10.1016/S1473-3099(18)30596-6 PMC6300482

[32] Gupta H, Srivastava S, Chaudhari S, et al. New molecular detection methods of malaria parasites with multiple genes from genomes. Acta Trop. 2016;160:15–22. 27130076 10.1016/j.actatropica.2016.04.013

[33] Siwal N, US S, Dash M, et al. Malaria diagnosis by PCR revealed differential distribution of mono and mixed species infections by Plasmodium falciparum and P. vivax in India. PLoS One. 2018;13:e0193046. 29565981 10.1371/journal.pone.0193046 PMC5863947

[34] Ghosh SK, Rahi M. Malaria elimination in India-The way forward. J Vector Borne Dis. 2019;56:32–40. 31070163 10.4103/0972-9062.257771

[35] Mohapatra MK, Padhiary KN, Mishra DP, et al. Atypical manifestations of Plasmodium vivax malaria. Indian J Malariol. 2002;39:18–25. 14686106

[36] Naik BS. Incidence of jaundice in Plasmodium vivax malaria: A prospective study in Moodabidri, South India. Malays J Med Sci. 2014;21:24–27. PMC4418110 25977618

[37] Anand AC, Ramji C, Narula AS, et al. Malarial hepatitis: a heterogeneous syndrome? Natl Med J India. 1992;5:59–62. 1304265

[38] Chawla LS, Sidhu G, Sabharwal BD, et al. Jaundice in Plasmodium falciparum. J Assoc Physicians India. 1989;37:390–391. 2687227

[39] Kochar DK, Agarwal P, Kochar SK, et al. Hepatocyte dysfunction and hepatic encephalopathy in Plasmodium falciparum malaria. QJM. 2003;96:505–512. 12881593 10.1093/qjmed/hcg091

[40] Kochar DK, Singh P, Agarwal P, et al. Malarial hepatitis. J Assoc Physicians India. 2003;51:1069–1072. 15260391

[41] Abdallah TM, Abdeen MT, Ahmed IS, et al. Severe Plasmodium falciparum and Plasmodium vivax malaria among adults at Kassala Hospital, eastern Sudan. Malar J. 2013;12:148. 23634728 10.1186/1475-2875-12-148 PMC3655045

[42] Zubairi AB, Nizami S, Raza A, et al. Severe Plasmodium vivax malaria in Pakistan. Emerg Infect Dis. 2013;19:1851–1854. 24188313 10.3201/eid1911.130495 PMC3837647

[43] Baird JK. Reinventing primaquine for endemic malaria. Expert Opin Emerg Drugs. 2012;17:439–444. 22998791 10.1517/14728214.2012.720252 PMC3514001

[44] Kumar P, Yadav U, Rai V. Prevalence of glucose-6-phosphate dehydrogenase deficiency in India: an updated meta-analysis. Egyptian J Med Human Gene. 2016;17:295–302.

[45] Mohanty D, Mukherjee MB, Colah RB. Glucose-6-phosphate dehydrogenase deficiency in India. Indian J Pediatr. 2004;71:525–529. 15226563 10.1007/BF02724295

[46] Mukherjee MB, Colah RB, Martin S, et al. Glucose-6-phosphate dehydrogenase (G6PD) deficiency among tribal populations of India - Country scenario. Indian J Med Res. 2015;141:516–520. 26139767 10.4103/0971-5916.159499 PMC4510748

[47] Mohanty D, Sukumar S, Mukherjee MB, et al. G6PD deficiency and malaria in India. Am J Hematol. 2003;72:150–151. 12555222 10.1002/ajh.10276

[48] Recht J, Ashley EA, White NJ. Use of primaquine and glucose-6-phosphate dehydrogenase deficiency testing: divergent policies and practices in malaria endemic countries. PLoS Negl Trop Dis. 2018;12:e0006230. 29672516 10.1371/journal.pntd.0006230 PMC5908060

[49] Anyanwu PE, Fulton J, Evans E, et al. Exploring the role of socioeconomic factors in the development and spread of anti-malarial drug resistance: a qualitative study. Malar J. 2017;16:203. 28521791 10.1186/s12936-017-1849-1 PMC5437569

[50] Berkman LF, Kawachi I, Glymour MM. Social epidemiology. Oxford, UK: Oxford University Press; 2014. https://oxfordmedicine.com/view/10.1093/med/9780195377903.001.0001/med-9780195377903

[51] Degarege A, Fennie K, Degarege D, et al. Improving socioeconomic status may reduce the burden of malaria in sub Saharan Africa: A systematic review and meta-analysis. PLoS One. 2019;14:e0211205. 30677102 10.1371/journal.pone.0211205 PMC6345497

[52] Tusting LS, Ippolito MM, Willey BA, et al. The evidence for improving housing to reduce malaria: a systematic review and meta-analysis. Malar J. 2015;14:209. 26055986 10.1186/s12936-015-0724-1 PMC4460721

[53] WHO World Health Organization. World Malaria Report. 2014.

[54] Fowkes FJ, Boeuf P, Beeson JG. Immunity to malaria in an era of declining malaria transmission. Parasitology. 2016;143:139–153. 26741253 10.1017/S0031182015001249

