# Supplementary material for: Clinical and epidemiological characterization of severe Plasmodium vivax malaria in Gujarat, India
Source: Virulence. 2020 Jun 3;11(1):730–8. doi: 10.1080/21505594.2020.1773107 (PMC7549892; doi:10.1080/21505594.2020.1773107)

**Clinical and epidemiological characterization of severe *Plasmodium vivax* malaria in Gujarat, India**

**Supplementary figure 1: Parasitemia, hemoglobin, white blood cells and platelet counts in falciparum and vivax malaria patients**. Parameters were compared between patients with *P. vivax* (*Pv*) and *P. falciparum* (*Pf*) infections. T bars represent median and Interquartile Ranges (IQR),y-axis is in log10,symbol star (*) represents levels of significance (* <0.05 and *** <0.001) and both asexual and sexual parasitemias were recorded by microscopy.


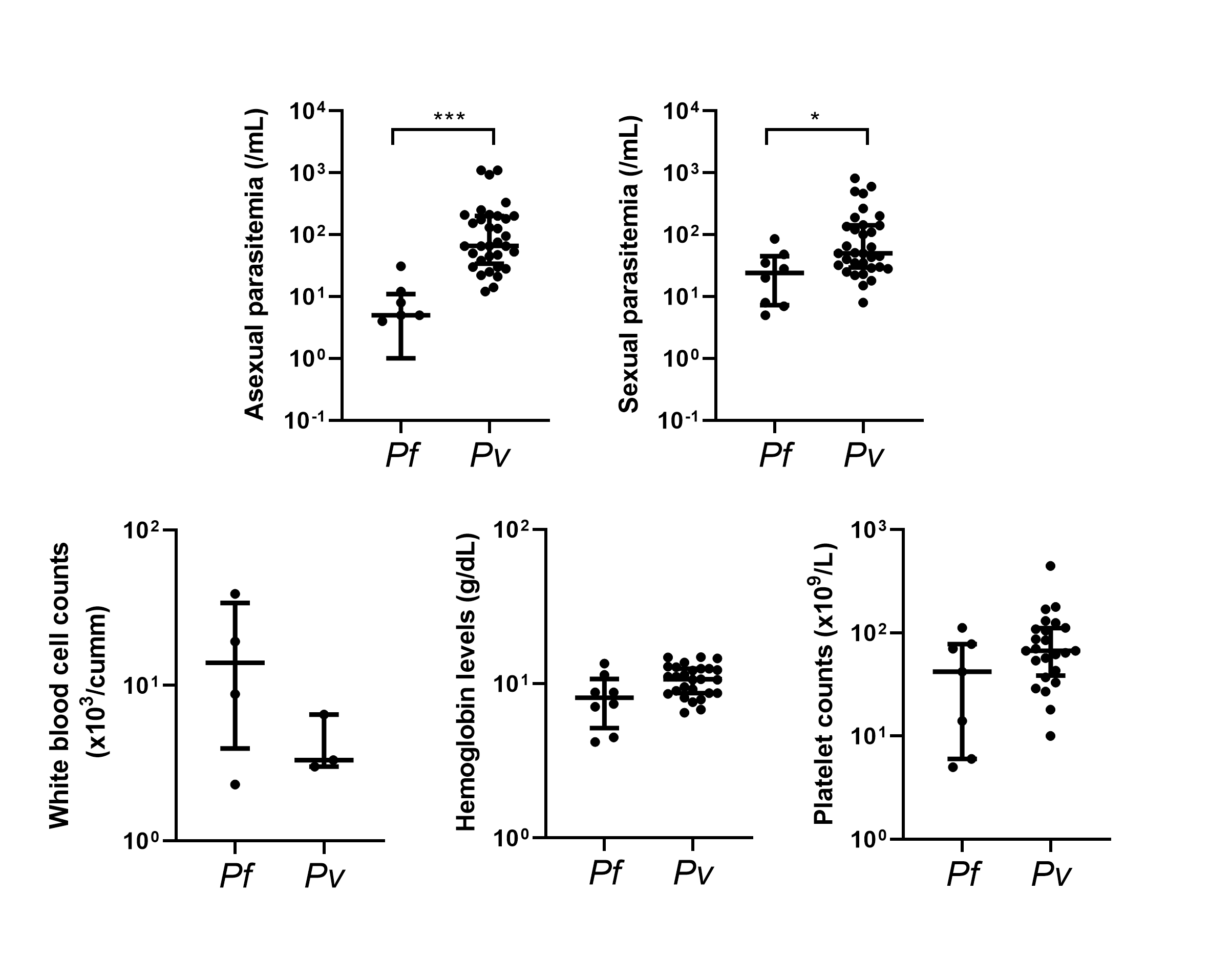

Supplement: Supplemental Material [file KVIR_A_1773107_SM2735.docx]
